# Comparative study of advanced hydrogen liquefaction using triple cascade mixed refrigerant cycles with integrated energy exergy economic and environmental analysis

**DOI:** 10.1038/s41598-025-14258-8

**Published:** 2025-09-01

**Authors:** M. Shawky Ismail, M. Abd ElSalam ElSeuofy, Abd ElHamid Attia, Wael M. El-Maghlany, Mohamed ElHelw

**Affiliations:** Mechanical Engineering Department, Faculty of Engineering, Alexandria University, Alexandria, 21544 Egypt

**Keywords:** Hydrogen liquefaction, Specific energy consumption, Optimization, Carbon footprint, Energy-exergy-economic analysis, Mechanical engineering, Energy infrastructure, Energy storage, Environmental sciences

## Abstract

Hydrogen, as a clean energy source, is recognized as a pivotal energy carrier in the global transition to sustainable energy systems and serves as a crucial pathway for energy storage and efficient utilization within cryogenic systems. Hydrogen liquefaction is one of the most promising methods for increasing its energy density, enabling more efficient storage, transportation, and utilization in large-scale energy systems. However, substantial challenges persist, particularly regarding the high energy consumption associated with the liquefaction process. This study addresses these challenges by proposing two designs for a triple-cascade mixed refrigerant cycle aimed explicitly at reducing energy consumption for high-density hydrogen storage: 66.7 kg/m^3^ at − 245 °C (Case 1) and 76 kg/m^3^ at − 249 °C (Case 2). The proposed systems utilize two mixed refrigerant cycles for the precooling and cryogenic stages. In Case 1, pure nitrogen is employed as the third refrigerant in the precooling stage, whereas Case 2 incorporates a regenerative cryogenic hydrogen cycle as the third refrigerant throughout the entire system, coupled with a carbon dioxide cycle for compressor cooling. Simulations were conducted using Aspen HYSYS, with optimization through the Aspen Optimizer algorithm. The results indicate that Case 1 achieves a specific energy consumption (SEC) of 6.98 kWh/kgH₂, representing a 17.4% reduction from the baseline, while Case 2 reduces SEC to 6.19 kWh/kgH₂, a 14.5% decrease. The exergy analysis of the heat exchangers shows a 37% reduction in exergy destruction in Case 2 compared to Case 1. Additionally, Case 2 demonstrates a 5.8% reduction in capital expenditure and a 22% reduction in carbon footprint (CFP). These findings highlight the potential of the proposed triple-cascade process to enhance energy efficiency, improve both thermodynamic and economic performance, and reduce environmental impact.

## Introduction

In the contemporary era, energy security and global warming are among humanity’s most pressing challenges. Tackling these issues is crucial for ensuring long-term environmental sustainability and energy resilience. The United Nations Sustainable Development Goals (SDGs)^1^ underscore the urgent need for innovative solutions and supportive policies to combat climate change and advance clean energy technologies. In this context, hydrogen is increasingly recognized as a pivotal energy carrier for sustainable development, with versatile applications across diverse sectors. Because hydrogen produces zero carbon emissions at the point of use, it plays an indispensable role in decarbonizing heavy industry and the petroleum sector^2^. The continued reliance on fossil fuels has exacerbated environmental degradation, particularly global warming, making the transition to renewable energy sources, such as hydrogen, a critical mitigation strategy. Boasting abundance, high energy density, and significant calorific value, hydrogen emerges as a promising solution^3^ to the global energy crisis. Shifting from fossil fuels to clean hydrogen is a key approach to lowering CO₂ emissions^4^ and bolstering energy security. Moreover, integrating hydrogen into energy systems supports SDG 7 (Affordable and Clean Energy) and SDG 13 (Climate Action), strengthening global sustainability efforts. Effective hydrogen storage solutions are vital to accelerate this transition and establish a more resilient, sustainable energy infrastructure.

Inherent to the widespread use of hydrogen as an energy carrier is its notably low volumetric energy density, approximately 0.00278 kW/m3 under ambient conditions^5^. Such a limitation introduces formidable challenges for long-term storage and long-haul transportation, particularly in applications where compact, high-energy–density fuels are essential^6^. To address this constraint, a variety of hydrogen storage technologies have been investigated, including high-pressure compressed gas, organic liquid carriers, physical adsorption media, and cryogenic liquid storage^7^. Among these, hydrogen liquefaction emerges as one of the most promising approaches, as it substantially increases volumetric energy density. Specifically, converting hydrogen to its liquid form raises the volumetric energy density to 2.36 kW/m3^8^. Furthermore, liquefaction improves volumetric efficiency: Liquid hydrogen (LH₂) occupies approximately one eight-hundredth of the volume of gaseous hydrogen at standard atmospheric pressure, thereby enhancing transport and storage efficiency. This reduction in volume offers an estimated six to eightfold increase in transport efficiency compared with compressed gas^9^. Accordingly, liquefied hydrogen presents a compelling option for large-scale energy applications, mitigating logistical constraints and facilitating practical deployment.

Hydrogen liquefaction constitutes a critical yet energy-intensive stage in the hydrogen supply chain, since its critical point occurs at approximately –240 °C at 13 bar. Although the minimum theoretical thermodynamic energy requirement for hydrogen liquefaction under ideal conditions is 2.9 kWh/kg^10^, actual SEC values are substantially higher, incurring significant energy consumption and operational costs, rendering liquefaction both capital- and energy-intensive. Contemporary hydrogen liquefaction facilities exhibit an SEC of approximately 13–15 kWh/kg, with exergy efficiencies of 20–30% below the critical point^11^. Given these constraints, there is a pressing imperative for advanced, high-efficiency liquefaction technologies to minimize energy costs and facilitate the widespread adoption of liquid hydrogen in energy markets^12^. This review examines significant advancements in hydrogen liquefaction and storage for large-scale processes over the last twenty years, with a focus on cooling strategies such as liquid nitrogen (LN₂), helium, mixed refrigerant cycles, and liquefied natural gas (LNG).

Berstad et al.^13^ explored an innovative hydrogen liquefier that employed mixed-refrigerant precooling followed by a reversed helium/neon Brayton cycle, achieving notable efficiency improvements. Their design reduced power consumption to 6.48 kWh/kg of liquid hydrogen. Similarly, Krasae-in^14^ optimized a large-scale liquid hydrogen plant by implementing a mixed-fluid refrigeration system with carefully selected refrigerant compositions and ortho–para hydrogen conversion, ultimately lowering power consumption to 5.91 kWh/kg LH₂. For a single mixed refrigerant cycle, Sadaghiani et al.^15^ proposed an advanced hydrogen liquefaction process, analyzing it through energy, exergy, and economic methodologies and reported a specific energy consumption of 7.64 kWh/kg LH₂. Chang and Park^16^ conducted a thermodynamic analysis of hydrogen liquefaction using cascade Joule–Thomson systems with single-component refrigerants, identifying optimized cycle configurations that achieve high figures of merit (FOM) without requiring expansion machines, thereby offering improved reliability and scalability for large-capacity applications. Particularly, Cardella et al.^17^ focused on hydrogen liquefaction costs by balancing capital and operating expenditures, estimating liquefaction costs ranging from $5 to $7 per kg LH₂ for the studied models. For the study of various refrigerants in a cryogenic system, Tan et al.^18^ conducted a comparative analysis of cryogenic energy storage systems using different refrigerants, such as nitrogen, argon, and methane as working media, and achieved the highest efficiency with methane. Ghorbani et al.^19^ proposed a novel integrated hydrogen purification and liquefaction system combining natural gas steam reforming, an organic Rankine cycle, and photovoltaic panels, achieving a specific energy consumption of 8.592 kWh/kg LH₂ and exergy efficiency of 72.41%, with further optimization reducing energy use to 7.881 kWh/kg LH₂ under enriched methane input.

Nevertheless, with reliance on liquid nitrogen (LN₂) in the precooling stage, Alekseev et al.^20^ provided a comprehensive overview of hydrogen liquefaction technologies, including precooling methods, particularly liquid nitrogen. Alekseev analyzed the efficiency and economic aspects of different liquefaction techniques, offering insights into the optimization of hydrogen liquefaction processes. In contrast, Cardella et al.^21^ sought to reduce dependency on liquid nitrogen due to concerns about economic viability, instead favoring a high-pressure hydrogen cycle combined with a mixed-refrigerant precooling system. To explore alternative refrigerants for sustainable and cost-effective hydrogen liquefaction, the use of liquefied natural gas (LNG) cold energy in hydrogen liquefaction has gained considerable attention. Kuendiga et.al ^22^. introduced a novel method that reduced power consumption by 11% compared to traditional approaches, leveraging the free cooling capacity of LNG. This was followed by work from Wilhelmsen et al.^23^ and He T et al.^24^, who optimized LNG integration into hydrogen liquefaction to further enhance energy efficiency and lower operational costs. Similarly, Cho et al.^25^ achieved a high reduction by integrating LNG cold energy into a base-case hydrogen liquefaction design comprising a two-stage mixed refrigerant (MR) precooling cycle and a cryogenic Joule–Brayton (J–B) cycle. This integration resulted in a specific energy consumption (SEC) of 4.07 kWh/kg LH₂. Chang et al.^26^ developed a hydrogen liquefaction process integrating LNG cold energy with a closed-cycle Brayton refrigeration system, identifying a two-stage expansion cycle with LNG precooling as optimal for minimizing power consumption and enabling compact, efficient operation in a 0.5 ton/day pilot system. On the other hand, at pressures above the critical point of hydrogen, supercritical hydrogen storage systems, as investigated by Song et al.^27^, were designed to produce high-density cryogenic hydrogen, achieving a specific energy consumption (SEC) of 5.432 kWh/kg H₂. Moreover, the study conducted by Xu et al.^28^ examined higher-density (70 kg/m3) supercritical hydrogen storage systems, addressing specific energy consumption (SEC) values of 6.422 kWh/kgH₂ for the DPMR process and 6.872 kWh/kgH₂ for the DCMR process. Consequently, the use of a portion of the hydrogen product as a refrigerant was presented by Ahmad et al.^29^. The study conducted a comparative energy and exergy analysis of hydrogen liquefaction processes with and without ortho-para hydrogen conversion. Using a cascaded five-stage Brayton refrigeration cycle simulated in Aspen Plus, they demonstrated that the ortho-parahydrogen conversion configuration significantly outperforms the non-conversion setup, with a specific energy consumption of 8.45 kWh/kgH₂ compared to 15.65 kWh/kgH₂. The use of hydrogen as a second refrigerant in a dual cycle with one mixed refrigerant was investigated by Tamarona et al.^30^. The author investigated the techno-economic feasibility of a hydrogen liquefaction process using the high-pressure Claude cycle, with the plant’s overall SEC determined to be 7.241 kWh/kgH₂. On the other hand, regarding the integration of cryogenic hydrogen with energy systems, Lenger et al.^31^ introduced a reversible cryogenic exergy utilization system for fuel cell systems, utilizing cryogenic hydrogen exergy to reduce heat rejection to ambient by 40–67% and lower power demand by 31%, outperforming conventional heat exchangers.

These pivotal studies have established a foundation for hydrogen storage research. The primary focus has been on successive enhancements to the fundamental hydrogen liquefaction process, emphasizing the introduction of sophisticated refrigeration cycles, systems integration, and process optimization. Nevertheless, these strategies frequently encounter considerable implementation barriers due to complex process design and the need for synergistic integration with ancillary processes, such as employing liquid nitrogen or liquefied natural gas for pre-cooling, which impose significant primary energy penalties. Additionally, research into chemical hydrogen conversion, including ortho-to-para hydrogen conversion, introduces additional thermal-management complexities and often relies on idealized assumptions concerning feed conditions, cooler temperatures, and hydrogen density, rendering these approaches susceptible to suboptimal minimum. Consequently, a clear gap persists in the literature for innovations that optimize energy efficiency, economic viability, and environmental sustainability. To address these challenges, this study proposes two advanced triple-cascade mixed-refrigerant (TCMR) configurations designed to enhance cycle performance, evaluate the impact of a third refrigerant, and minimize specific energy consumption, thereby enabling high-density hydrogen liquefaction below the critical point.

## Methodology

**Fig. 1:**
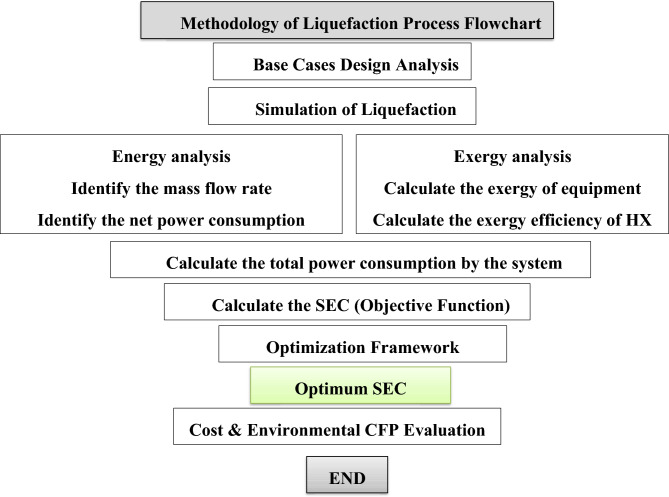
Methodology adopted for system analyses in the study.

The innovative Triple Cascade Mixed-Refrigerant (TCMR) cycle represents a groundbreaking approach to hydrogen liquefaction, incorporating several key advancements: introducing a third refrigerant, nitrogen in Case 1 and hydrogen in Case 2, to the conventional dual-cascade cycle; optimizing mixed-refrigerant compositions; integrating a decarbonization cycle; and standard equipment design to yield a high-density product. Moreover, Case 1 is benchmarked against the selected base case of the dual cycle (DCMR-OP ^29^, while Case 2 is compared to the dual cycle (DCMR-H_2_)^30^, to quantify improvements in energy demand and overall system performance. This study focuses on optimizing the TCMR cycle cases by targeting critical energy-use factors and refining physical parameters such as pressure and temperature using Aspen Optimizer V.11. Furthermore, a genetic algorithm fine-tunes refrigerant compositions to minimize specific energy consumption, delivering significant process enhancements. In Case 2, the incorporation of a CO₂-based decarbonization cycle enhances compressor cooling efficiency, further reducing energy demands. Comprehensive thermodynamic, exergy, and techno-economic analyses validate the system’s efficiency, feasibility, cost-effectiveness, and environmental impact for both cases. The methodology flowchart is shown in Fig. 1.

## Process description and model development

The proposed Triple Cascade Mixed Refrigerant (TCMR) cycle builds upon the conventional dual-cycle configuration by incorporating key modifications and enhancements. The process is systematically divided into two principal stages: precooling and deep-cooling, which are further subdivided into four distinct loops: the hydrogen product cycle (H), the mixed-refrigerant pre-cooling cycle (M), the mixed-refrigerant deep-cooling cycle (R), and the tertiary refrigerant loop: nitrogen (N) in Case 1 and hydrogen refrigerant (HF) in Case 2.

### Process description case (1)

**Fig. 2:**
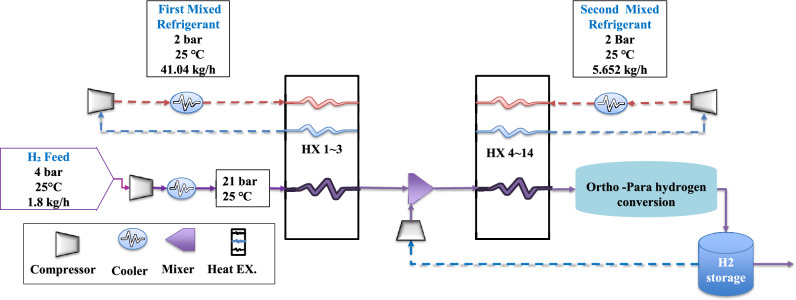
The flowchart of DCMR-OP.

The process description of the selected dual-cycle base study^29^ features the integration of a hydrogen regenerative-return system within the deep-cooling cycle and includes ortho–para hydrogen conversion. Figure 2 presents a simplified flowchart of the DCMR-OP system.

Figure 2 illustrates that feed hydrogen is first compressed to 21 bar and cooled to 25 °C^29^. Thereafter, in the pre-cooling section, it is further cooled to − 190 °C via a series of multi-stream heat exchangers. In the deep-cooling stage, hydrogen is reduced to − 245 °C through additional heat exchangers combined with ortho–para hydrogen conversion. Two mixed-refrigerant loops facilitate these cooling stages: the first blend comprises methane (17%), propane (18%), n-pentane (15%), ethane (7%), nitrogen (17%), R14 (8%), butane (2%), and ethylene (1.6%), while the deep-cooling loop utilizes predominantly helium (89.8%) supplemented by neon (10.2%).

**Fig. 3:**
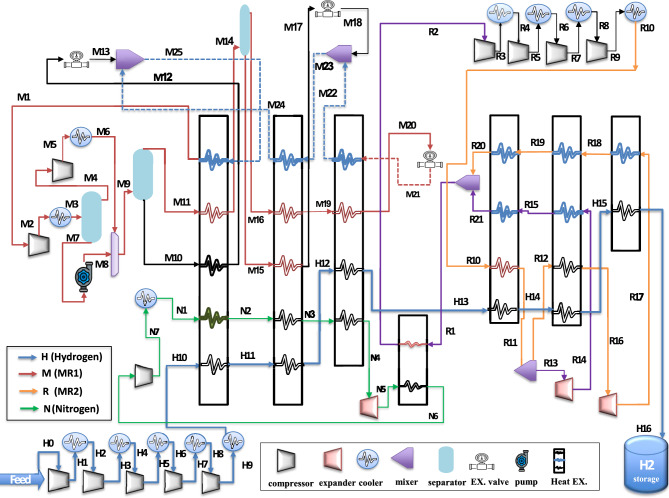
The proposed cycle of TCMR flow diagram Case 1.

The proposed Case 1 process flowchart of the TCMR cycle, demonstrating the integration of the triple refrigerant cycle based on the conditions of the base cycle^29^ without ortho-parahydrogen conversion, is illustrated in Fig. 3. This figure provides a clear depiction of the operational framework of the TCMR cycle.

The flow diagram for Case 1 in Fig. 3 is divided into four cycles: the hydrogen product (H), first mixed refrigerant (M), second mixed refrigerant (R), and third refrigerant (N). The hydrogen feed, initially at 4 bar, undergoes a five-stage compression process with intercooling stages, reaching 21 bar and 35 °C at (H10). The hydrogen is then cooled to –141.5 °C at (H13) through a series of multi-flow heat exchangers (HX1-HX3) in the pre-cooling section. In the subsequent deep-cooling section, the hydrogen is further cooled to –245 °C through multi-flow heat exchangers (HX4-HX6). This process results in the production of cryogenic liquid hydrogen for storage.

The first mixed refrigerant cycle (M) in the pre-cooling section, initially at (M1), is compressed, cooled, and separated into vapor and liquid streams. These streams are pressurized, cooled, and recombined at (M9) (9.85 bar, 35 °C) before entering a vapor–liquid separator. The liquid stream (M10) and vapor stream (M11) are then directed to the heat exchanger HX1. The liquid stream (M12) is throttled to 1.5 bar and combined with the return stream to provide cooling duty to HX1 via (M25). Meanwhile, the vapor stream (M11) is cooled and separated into (M15, M16), which then enter HX2. The liquid stream (M15) is cooled, throttled, and merged with the return line (M22) to provide cooling duty for HX2 at (M23). The vapor stream (M16) is cooled through HX2 and HX3 to (M19) and throttled to provide cooling duty for HX3 at (M21), where continuous cooling and compression occur in a closed cycle.

The second mixed refrigerant cycle (R) in the deep-cooling section is initially compressed at (R1) through four-stage compressors, reaching 23.9 bar and 35 °C at (R10). The refrigerant is further cooled in HX4 at (R11) and splits into two streams: one expands to 1.8 bar for HX5 cooling duty, while the other is cooled to –206.7 °C at (R16), which is then expanded to –246.7 °C to provide cooling duty for HX6. Finally, the cooled mixtures are recycled to maintain continuous operation in a closed cycle.

The third refrigerant cycle (N) involves nitrogen (N1), which is compressed to 3 bar, cooled to 35 °C, and passes through HX1 to HX3, where it is cooled to -37.4 °C at (N4). The nitrogen is then expanded to –51.2 °C at (N5) to provide cooling to the inlet line of a compressor at (R1), lowering the temperature to 19.2 °C at (R2). The nitrogen then returns to being compressed and cooled as part of the closed cycle. The key difference between the dual cycle^29^ and the triple cycle lies in the unique TCMR design. The TCMR system uses seven heat exchangers instead of fourteen, incorporates a third refrigerant to enhance heat transfer and energy efficiency, eliminates reliance on parahydrogen reactors, and optimizes the mixed refrigerant composition.

### Process description case (2)

**Fig. 4:**
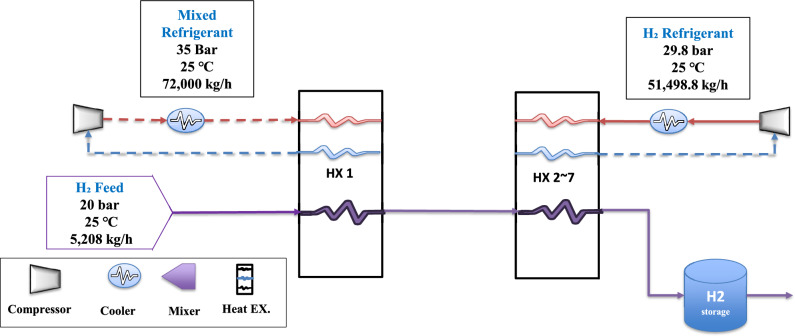
The flow diagram of (DCMR-H_2_).

The second selected reference^30^ investigates the utilization of hydrogen as an auxiliary refrigerant in the liquefaction cycle. This approach employs a single mixed refrigerant, and its simplified process flow diagram is depicted in Fig. 4 (DCMR-H₂).

In this configuration (Fig. 4), the hydrogen feed enters at 20 bar and 25 °C ^30^. It then undergoes precooling by a mixed refrigerant to − 159 °C via a multi-stream heat exchanger. The mixed refrigerant comprises methane (32.4%), propane (3.1%), ethane (27.4%), nitrogen (10.1%), and n-butane (27%). During the deep-cooling stage, the hydrogen refrigerant cools the hydrogen product to − 243 °C at 20 bar using additional multi-stream heat exchangers, before throttling to 1.5 bar to yield LH₂ at − 252 °C.

**Fig. 5:**
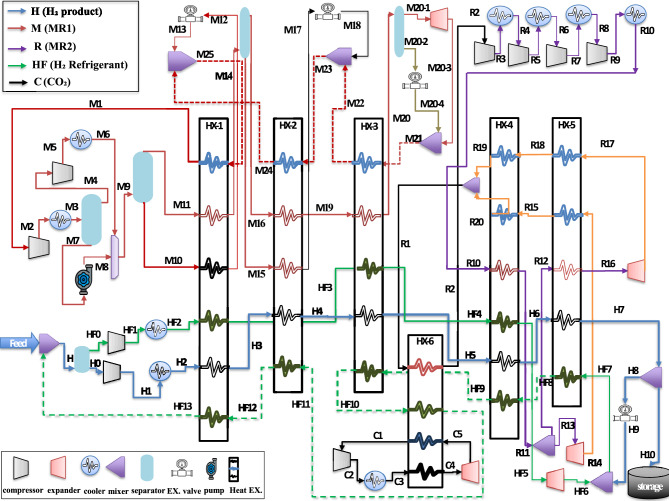
The proposed cycle of TCMR flow chart Case 2.

The proposed process flowchart for Case 2 of the TCMR cycle, which illustrates the use of hydrogen as the third refrigerant, is shown in Fig. 5. This figure provides a clear representation of the operational framework and process flow for Case 2.

As shown in Fig. 5, the hydrogen product feed (H) at 21 bar undergoes single-stage compression with cooling, reaching 47.7 bar at 35 °C. It is then cooled to − 155 °C using multi-flow heat exchangers (HX1–HX3) in the precooling section. In the deep-cooling section, additional heat exchangers (HX4–HX5) further cool it to − 249 °C, producing cryogenic liquid hydrogen for storage.

In the first mixed refrigerant cycle (M) for the precooling section, the refrigerant undergoes compression, cooling, separation, and throttling, as detailed in Case 1. The second refrigerant cycle (R) for the deep-cooling section begins with the initial compression and cooling of (R2) to 35 °C and 26.9 bar at point (R10). The compressed refrigerant is then further cooled using heat exchangers HX4 and HX5. After exiting HX4, the cooled (R11) splits into two streams: one stream expands to 1.8 bar at point (R14), generating subcooling for HX5, while the other stream passes through HX5 and is cooled to − 213.6 °C at point (R16). In subsequent deep-cooling stages, the (R16) stream passes through an additional expander, reducing its temperature to − 250.3 °C to meet the final cooling requirements of HX5. The cooled mixtures are then recycled through the system for continuous cooling and compression.

The third refrigerant, hydrogen (HF), initially undergoes single-stage compression with cooling, reaching 24.7 bar and 35 °C at (HF2). It is then further cooled to − 85.6 °C at (HF4) through multi-flow heat exchangers HX1 and HX3 in the precooling section. In the deep-cooling section, the hydrogen is cooled at (HF5) in HX4, then expanded at (HF6) to − 200.3 °C. At this critical point, it is mixed with a portion of the liquid hydrogen product (H9) to provide a highly cooled refrigerant at HF7 at − 206.4 °C. Hence, this cold stream plays three significant roles in the whole cycle. Firstly, it acts as a reverse refrigerant to provide additional cooling duty in the deep-cooling section (HX5 and HX4), where it is heated to − 127.9 °C at (HF9). Secondly, it provides extra cooling duty for HX3 in the precooling section, reaching − 27.9 °C at (HF10). Thirdly, it is responsible for cooling the carbon dioxide refrigerant (C) in HX6, and it finally returns to the mainstream feed hydrogen.

Finally, the cycle involves the carbon dioxide (CO₂) refrigerant (C), which undergoes single-stage compression with cooling, reaching 4 bar at (C3). The CO₂ then passes through heat exchanger HX6, cooling to − 26 °C, and is further cooled to − 79.6 °C via an expander at (C5). This cooled CO₂ is used to lower the inlet stream temperature of (R1) from 34 °C to 18 °C at (R2) before the compression stage.

The primary distinctions between the conventional dual-cycle hydrogen refrigeration system and the proposed TCMR cycle include: the introduction of a tertiary hydrogen refrigerant loop; inlet cooling of the refrigerant compressor; an optimized mixed-refrigerant formulation; integration of a CO₂-based decarbonization cycle; and the production of high-density liquid hydrogen for storage.

### Process modeling

Upon finalizing the initial process design, the selection of optimal parameters and operating conditions is paramount. In this work, Aspen HYSYS V.11^32^ is employed to model the proposed TCMR cycle, implementing the Peng–Robinson equation of state^33^ due to its proven accuracy in predicting thermodynamic behavior across diverse substances. To reflect realistic engineering practice, the following literature-based assumptions are adopted:

I. The system operates under steady-state conditions, with kinetic and potential energy effects assumed negligible^28,34^.
II. Hydrogen gas enters the process at 25 °C.
III. Pressure drops across water coolers and heat exchangers are neglected^28,35^.
IV. Pressure drops in separators and other devices are ignored^35,36^.
V. The outlet temperature of all water coolers is maintained at 35 °C^28^.
VI. The adiabatic efficiencies of all compressors and the pump are set at 80%, while the expanders operate at 90%^13,28,34^.
VII. A minimum temperature difference greater than 1 °C is assumed in all heat exchangers^28,34^.

The assumptions in this analysis derive from standard steady-state hydrogen liquefaction process properties^34^. They streamline the simulation and are consistently applied across numerous recent studies^29,36^ and to all cases, ensuring uniform influence on process performance.

### Process optimization

Process optimization is paramount following initial design and simulation. At this stage, enhancing thermodynamic performance and curbing energy consumption become imperative. Accordingly, the Aspen HYSYS optimization module is employed to establish a comprehensive optimization framework. In previous studies, the complex non-linear problem of the hydrogen liquefaction process in HYSYS has typically been optimized using genetic algorithms (GA)^37^. The server in HYSYS can link the optimizer code to achieve the purpose of finding the optimal solution for the liquefaction process^36^. The optimizer leverages Sequential Quadratic Programming (SQP) as its core algorithm. This framework targets the mole fractions of mixed refrigerant (R) to minimize overall specific energy consumption. Simultaneously, critical thermodynamic parameters, including mixed refrigerant temperatures, pressures, and mass flow rates, are optimized to improve system efficiency further.

## Performance models

### Performance relative to base cases

The performance evaluation contrasts the Triple Cascade Mixed Refrigerant (TCMR) cycle configurations (Case 1 and Case 2) against the base-case configurations, DCMR-OP and DCMR-H₂, under identical operating conditions. To quantify the proposed TCMR cycle’s effectiveness and efficiency, a detailed analysis of key performance indicators was conducted. The primary metrics include hydrogen feed pressure, hydrogen feed temperature, liquid hydrogen (LH₂) product pressure and temperature, LH₂ density, and the outlet temperature of the cooler. All configurations were simulated under the same inlet operational conditions to ensure a fair comparison and to evaluate the impact of transitioning from a dual-cycle to a triple-cycle configuration.

### Energy and exergy balance

To assess the thermodynamic performance of the TCMR systems, governing balance equations were applied to calculate parameters such as enthalpies and exergies, thereby establishing a framework for rigorous analysis. The principal balance equations employed are outlined below, covering mass conservation, energy conservation, and exergy analysis, each customized to the relevant process streams. Collectively, these equations underpin a detailed comparative evaluation of both systems. A central performance metric is the Specific Energy Consumption (SEC), quantifying system efficiency as the ratio of total energy consumption to hydrogen mass flow rate; its defining equation^38^ is presented below in Eq. 1:

1 $$SEC= \frac{{W}_{net}}{{m}_{product}}= \frac{\left(\sum {W}_{COMP} + \sum {W}_{PUMP} - \sum {W}_{EXP}\right)}{{m}_{{H}_{2}}}$$

Additionally, the governing equation of Figure of Merit (FOM) assesses system performance by comparing actual energy use to the theoretical minimum requirement in Eq. 2:

2 $$FOM = \frac{{W_{rev} }}{{W_{act} }} = \frac{{m_{{H_{2} }} (\dot{E}_{{\Pr oduct_{{H_{2} }} }} - \dot{E}_{{feed_{{H_{2} }} }} )}}{{\left( {\sum W_{COMP} + \sum W_{PUMP} - \sum W_{EXP} } \right)}}$$

The general governing equation for energy balance of each system equipment is presented below in Eq. 3.

3 $$W= m ({h}_{o}- {h}_{i})$$

Since the proposed processes involve no chemical reactions and negligible differences in kinetic and potential energy, only physical exergy is considered. The rate of entropy generation (Ṡ_gen_) is a fundamental parameter that directly correlates with irreversibilities in the process. The formulas for exergy-destruction rate, the rate of entropy generation Ṡ_gen_, and the general exergy balance equation are listed below in Eqs. 4–8:

4 $$\dot{E}=({h}_{i}-{h}_{ref})-{T}_{ref}({s}_{i}-{s}_{\text{ref }})$$

5 $${\dot{S}}_{ gen}=m \left({s}_{o}-{s}_{i}\right)$$

6 $$\dot{E}={(\dot{E}}_{i}- {\dot{E}}_{O}) \pm W$$

where $\pm W$ *is* + *for compressors, for expanders and 0 for coolers*

7 $${\dot{E}}_{sup}=\sum m{\dot{E}}_{i}$$

8 $${\eta }_{ex}=1-\frac{\dot{E}}{{\dot{E}}_{sup}}$$

### Composite curve evaluation

Composite curve analysis constitutes a fundamental methodology for characterizing energy flows in multi-stream heat exchange systems by graphing composite hot and cold streams and quantifying the temperature difference between composite curves (TDCC) in relationship to temperature and heat flow. This matching technique serves as a critical tool for assessing heat transfer effectiveness, where the minimum gap between curves correlates with exergy destruction. Minimizing this temperature gap reduces irreversibilities and lowers energy consumption. Through systematic adjustment of design variables, such as temperature, flow rates, and heat exchanger area, the composite curves can be aligned more closely, resulting in an energy-efficient configuration and enhanced overall system performance.

### Economic evaluation model

Economic assessment is essential for projecting the cost structure of a commercial-scale liquefaction plant and evaluating the viability of the liquefaction cycle. Accordingly, a simplified economic-cost model is formulated, focusing on capital expenditures (CAPEX) and operational expenditures (OPEX). The CAPEX component encompasses the purchase costs of major process equipment, including compressors, expanders, multi-stream heat exchangers, water coolers, and auxiliary systems. Conversely, the OPEX model is primarily determined by energy costs, although labor and service expenses also contribute. For this study, OPEX is restricted to energy consumption owing to its preponderant influence. The model relies on the following assumptions:

I. The cost of the multi-stream heat exchanger is derived from the heat exchange surface area (m2). The heat transfer coefficient for each heat exchanger is assumed to be 110 W/m2^39^.
II. The cost of auxiliary systems, including piping, valves, and ancillary components, is estimated using a scaling factor applied to the total cost of primary equipment.
III. OPEX includes electricity costs, calculated based on the rate at which electrical energy is billed per kilowatt-hour (kWh) per year. As of March 2024, the global average electricity price for industrial use is approximately 15.2 U.S. cents per kWh^40^.
IV. The plant is assumed to operate 7,920 h per year.

These assumptions provide a concise yet robust framework for assessing the economic performance of the liquefaction process under the two operational scenarios, based on the calculations in the following equations Eq. 9–16:

Cost Formula^21,41–43^,

9 $$P{C}_{COMP}= 11720 {\left(\frac{{W}_{COMP}}{0.7355}\right)}^{0.61}$$

10 $$P{C}_{Exp}= 632.4 {\left(\frac{{W}_{EXP} }{0.7355 }\right)}^{0.81}$$

11 $$P{C}_{PUMP}=2100 \left({\left(\frac{{W}_{PUMP}}{10}\right)}^{0.26}\right)\left(\frac{\left(1 - {\eta }_{PUMP}\right)}{{\eta }_{PUMP}}\right)$$

12 $$P{C}_{HX} =8500 + 409 {A}^{0.8}$$

13 $$P{C}_{WC} = 1773 ({m}_{H}+ {m}_{M} + {m}_{R} + {m}_{N,HF})$$

14 $$P{C}_{Other} = 0.18 (P{C}_{COMP} +P{C}_{EXP}+ P{C}_{PUMP} + P{C}_{HX} + P{C}_{WC})$$

15 $$CAPEX = (P{C}_{COMP} + P{C}_{EXP} + P{C}_{PUMP} + P{C}_{HX} + P{C}_{WC} + P{C}_{Other})$$

16 $$OPEX = (\$/kW) electricity cost \times \tau \times {W}_{COMP}$$

### Environmental and sustainability assessment

CO₂ emissions are universally acknowledged as the principal driver of anthropogenic climate change. Hydrogen liquefaction is associated with significant environmental burdens, notably greenhouse gas (GHG) emissions. This investigation evaluates two operation scenarios, Case 1 and Case 2, with an emphasis on reducing GHG and improving the process’s sustainability. In Case 2, a CO₂-based decarbonization cycle is incorporated into the liquefaction process to mitigate emissions. Through the systematic optimization of thermophysical parameters, adjustment of refrigerant blends, and inclusion of nitrogen or hydrogen as a tertiary refrigerant to lower energy consumption, the present approach achieves both emission reduction and enhanced alignment with the United Nations Sustainable Development Goals (SDGs). The key sustainability-oriented advancements and strategies explored in this study include:

- Reducing energy consumption via a cryogenic triple-cascade mixed-refrigerant cycle to minimize the liquefaction process’s environmental footprint.
- Performing a comprehensive carbon-footprint assessment, covering Scope 1 and Scope 2 emissions based on14064-1:2018^44^ guidelines, to quantify and monitor GHG releases from the liquefaction system.
- Utilizing captured CO₂ as a working refrigerant in Case 2 to promote carbon reuse and limit atmospheric emissions.
- Implementing a closed-loop cooling network to drastically curtail water consumption and advance resource conservation.
- Incorporating recycling protocols and employing the hydrogen product as a refrigerant to embody circular-economy principles within the liquefaction cycle.
- Recommending the integration of renewable energy sources for process power supply to support the sustainable generation of green hydrogen.

Scope 1 emissions encompass direct GHG emissions from the hydrogen liquefaction system, including fugitive refrigerant leaks. As reported by the Intergovernmental Panel on Climate Change (IPCC)^45^, annual average fugitive leakage rates for industrial refrigeration and cryogenic systems typically range from 7 to 10%; accordingly, a conservative value of 10% is adopted in this study to ensure a robust CFP assessment. Scope 2 emissions derive indirectly from purchased electricity, predominantly generated by coal or natural-gas-fired power plants. Within the hydrogen liquefaction context, these emissions are largely attributable to the energy consumption of compressors and pumps. The CO₂ emissions associated with electricity consumption can be quantified as follows in in Eq. 17:

17 $$e=\frac{\left({W}_{COMP}+{W}_{PUMP}\right)*\tau *\alpha }{{10}^{6}}$$

where (e) represents the mass of CO_2_ emissions in tonne, ($${W}_{COMP}+{W}_{PUMP})$$ represent the power consumed by the compressor and pump, respectively in kW, (τ) is operational period in hours, and ($$\alpha )$$ is the CO_2_ emission factor for coal-fired power plants, which is 877 g CO₂/kWh^46^ and 490 g CO₂/kWh for natural gas power plants^47^ according to IPCC. This emission factor reflects the amount of CO_2_ released per kilowatt-hour of electricity consumed from coal-based sources. Upstream (Scope 3) emissions, such as CO₂ capture and refrigerant production, are excluded to focus on process-internal variables and ensure consistent data quality.

CO₂ is categorized as an A1 refrigerant by the American Society of Heating, Refrigerating and Air-Conditioning Engineers (ASHRAE), meaning it is non-toxic and non-flammable under standard conditions. This makes it safer than many synthetic refrigerants^48^. CO₂ is considered an environmentally friendly refrigerant due to its Global Warming Potential (GWP) of 1 and its zero-ozone depletion potential, making it a viable alternative to refrigerants with higher environmental risks. However, despite its low direct contribution to global warming, the overall environmental impact of CO₂ refrigeration systems must also account for the energy consumption associated with their operation, as calculated in case 2. The integration of CO₂ as a refrigerant^49^ involves higher CAPEX due to specialized components, while OPEX can increase in warmer climates due to additional energy needs. The long-term feasibility of CO₂ depends on its availability, with methods like direct air capture and carbon capture and utilization being explored for sustainable use.

## Results and discussion

The proposed process optimization’s outcomes are presented and systematically compared with the two reference designs under identical design conditions and energy consumption metrics. Subsequent subsections detail analyses of energy efficiency, exergy performance, composite curve integration, economic evaluation, and environmental impact.

### Optmization results

The optimization of process design variables, including pressure, temperature, refrigerant flow rate, and the composition of the mixed refrigerant, was conducted to minimize the system’s specific energy consumption (SEC) and achieve the optimal objective function. Table 1 presents the upper and lower bounds for the optimization parameters and their corresponding optimal values for both cases. Table 2 identifies the constituent components of each refrigerant cycle.

**Table 1:** Upper and lower bounds.

Stream name	Variable	Unit	Lower bound	Optimal value	Upper bound	Stream name	Variable	Unit	Lower bound	Optimal value	Upper bound
Case 2						Case 1					
H1	Pressure	bar	21	47.7	50	H1	Pressure	bar	4	5.75	6
H3	Temperature	°C	-33	-29.58	34	H3	Pressure	bar	6	7.75	9
H4	Temperature	°C	-110	-90.63	-20	H5	Pressure	bar	9	10.82	13
H5	Temperature	°C	-166	-155.00	-110	H7	Pressure	bar	13	15.7	17
H6	Temperature	°C	-206	-190.00	-166	H9	Pressure	bar	17	21	22
H5	Temperature	°C	-250.3	-249.00	-206	H11	Temperature	°C	-36	-28.69	34
M1	Mass Flow	kg/h	2000	2420.0	3000	H12	Temperature	°C	-100	-82.73	-36
M2	Pressure	bar	1.5	4.7	6	M1	Mass Flow	kg/h	2000	2722	3000
M5	Pressure	bar	6	11	12	M2	Pressure	bar	1.5	4.7	6
M8	Pressure	bar	6	10	12	M5	Pressure	bar	6	9.85	2
M1	Temperature	°C	-33	25	34	M8	Pressure	bar	6	9.85	2
M12	Temperature	°C	-33	-25.21	34	M12	Temperature	°C	-36	-25.42	34
M17	Temperature	°C	-110	-100.30	-33	M17	Temperature	°C	-110	-100.6	-36
M19	Temperature	°C	-110	-109.00	-33	M20	Temperature	°C	-190	-187.6	-110
M20	Temperature	°C	-166	-150.00	-110	R1	Mass Flow	kg/h	1000	1207	1500
R1	Mass Flow	kg/h	1000	1170.00	1500	R12	Mass Flow	kg/h	700	884.31	900
R12	Mass Flow	kg/h	450	579.00	600	R3	Pressure	bar	1.8	3.53	5
R3	Pressure	bar	1.8	3.54	5	R5	Pressure	bar	5	6.92	8
R5	Pressure	bar	5	6.96	8	R7	Pressure	bar	8	13.57	17
R7	Pressure	bar	8	13.68	17	R9	Pressure	bar	17	26	28
R9	Pressure	bar	17	25	26	R11	Temperature	°C	-190	-165.6	-110
R11	Temperature	°C	-185	-161.40	-110	R16	Temperature	°C	-230	-206.6	-190
R15	Temperature	°C	-206	-175.00	-185	R18	Temperature	°C	-246.6	-237.6	-230
R18	Temperature	°C	-250.3	-198.00	-206	N1	Mass Flow	kg/h	500	700	750
HF0	Mass Flow	kg/h	25	50	90	N2	Temperature	°C	-25	-15.25	34
HF1	Pressure	bar	20	24.7	50	N3	Temperature	°C	-36	-30.50	-25
HF3	Temperature	°C	-33	-15	34	R1	He mole fraction	%	80	87.97	90
HF4	Temperature	°C	-110	-85.60	-33	R1	Neon mole fraction	%	10	12.03	15
HF13	Temperature	°C	-33	33.87	35						
C1	Mass Flow	kg/h	300	430.00	500						
R1	He mole fraction	%	80	86.96	90						
R1	Neon mole fraction	%	10	13.04	15

**Table 2:** Refrigerant compositions (mole %

Component	Mole fraction (%)	
	Case 1	Case 2
Component of the first refrigerant (M)		
Methane	22.2%	22.2%
Ethane	11.5%	11.5%
Propane	16.6%	16.6%
N-Pentane	23.8%	23.8%
Nitrogen	11%	11%
Ethylene	14.9%	14.9%
Component of the second refrigerant (R)		
Helium	87.97%	86.96%
Neon	12.03%	13.04%
Component of the third refrigerant (N)- (HF)		
Nitrogen	100%	0%
Hydrogen	0%	100%

The detailed outputs, including the pressure, temperature, flow rates, and exergy of each stream, are presented in Table 3 for both Case 1 and Case 2. These results provide a comprehensive view of the system’s performance under the optimized conditions.

**Table 3:** The result data of Case 1 and Case 2.

Case 2					Case 1				
stream	*m*	*Φ*	*T*	*P*	stream	*m*	*Φ*	*T*	*P*
Unit	kg/h	kJ/kg	°C	bar	Unit	kg/h	kJ/kg	°C	bar
FEED	100.00	3677.96	35.00	20.00	H0	100.00	1689.71	25.00	4.00
H	155.00	3677.76	34.60	20.00	H1	100.00	2174.42	66.69	5.75
HO	105.00	3677.76	34.60	20.00	H2	100.00	2136.70	25.00	5.75
H1	105.00	5032.83	145.67	47.70	H3	100.00	2530.05	58.98	7.75
H2	105.00	4759.08	35.00	47.70	H4	100.00	2506.90	35.00	7.75
H3	105.00	4838.55	-29.58	47.70	H5	100.00	2968.53	74.44	10.82
H4	105.00	5197.99	-90.63	47.70	H6	100.00	2918.56	35.00	10.82
H5	105.00	6161.38	-155.00	47.70	H7	100.00	3377.22	74.15	15.07
H6	105.00	7259.95	-190.00	47.70	H8	100.00	3327.79	35.00	15.07
H7	105.00	13,730.78	-249.00	47.70	H9	100.00	3788.03	74.24	21.00
H8	5.00	13,730.78	-249.00	47.70	H10	100.00	3738.43	35.08	21.00
H10	100.00	13,730.78	-249.00	47.70	H11	100.00	3814.20	-28.69	21.00
H9	5.00	13,327.42	-248.29	25.00	H12	100.00	4104.10	-82.73	21.00
HF-0	50.00	3677.76	34.60	20.00	H13	100.00	4843.82	-141.91	21.00
HF1	50.00	3962.94	59.11	24.70	H14	100.00	6123.96	-189.65	21.00
HF2	50.00	3939.56	35.01	24.70	H15	100.00	10,163.97	-235.56	21.00
HF3	50.00	3979.21	-15.00	24.70	H16	100.00	12,580.02	-245.00	21.00
HF4	50.00	4329.58	-85.60	24.70	M1	2722	24.74	25.00	1.50
HF5	50.00	6649.42	-196.93	24.70	M2	2722	105.58	82.10	4.79
HF6	50.00	6589.33	-200.39	21.00	M3	2722	95.60	35.00	4.79
HF7	55.00	6905.37	-206.48	21.00	M4	2476.4	101.25	35.00	4.79
HF8	55.00	6021.82	-186.96	21.00	M5	2476.4	152.22	73.27	9.85
HF9	55.00	4610.78	-127.90	21.00	M6	2476.4	139.79	35.00	9.85
HF10	55.00	3810.12	-27.36	21.00	M7	245.53	4.56	35.00	4.79
HF11	55.00	3740.47	38.83	21.00	M8	245.53	5.38	35.30	9.85
HF12	55.00	3739.71	37.58	21.00	M9	2722.0	130.59	35.55	9.85
HF13	55.00	3737.90	33.87	21.00	M10	921.50	15.86	35.55	9.85
M1	2420.00	24.74	25.00	1.50	M11	1800.5	170.30	35.55	9.85
M2	2420.00	104.20	81.19	4.70	M12	921.50	25.64	-25.42	9.85
M3	2420.00	94.66	35.00	4.70	M13	921.50	23.39	-28.50	1.50
M4	2224.33	99.62	35.00	4.70	M14	1800.5	199.79	-36.05	9.85
M5	2224.33	159.99	80.04	11.00	M15	875.46	115.44	-36.05	9.85
M6	2224.33	144.15	35.00	11.00	M16	925.04	236.84	-36.05	9.85
M7	195.67	4.40	35.00	4.70	M17	875.46	174.09	-100.60	9.85
M8	195.67	5.26	35.31	10.00	M18	875.46	171.80	-101.20	1.50
M9	2420.00	131.00	33.75	10.00	M19	925.04	387.92	-97.50	9.85
M10	864.33	16.82	33.75	10.00	M20	925.04	808.09	-187.60	9.85
M11	1555.67	173.65	33.75	10.00	M21	925.04	803.78	-188.60	1.50
M12	864.33	26.67	-25.21	10.00	M22	925.04	191.31	-107.63	1.50
M13	864.33	24.29	-28.69	1.50	M23	1800.5	200.04	-101.60	1.50
M14	1555.67	201.26	-33.94	10.00	M24	1800.5	62.31	-41.34	1.50
M15	708.63	113.68	-33.94	10.00	M25	2722.0	61.33	-37.05	1.50
M16	847.04	235.57	-33.94	10.00	N1	700.00	22.21	35.00	1.30
M17	708.63	173.04	-100.30	10.00	N2	700.00	25.14	-15.25	1.30
M18	708.63	170.72	-100.84	1.50	N3	700.00	28.17	-30.50	1.30
M19	847.04	429.36	-109.00	10.00	N4	700.00	29.95	-37.47	1.30
M20	847.04	617.84	-150.00	10.00	N5	700.00	11.06	-51.29	1.00
M21	847.04	591.51	-166.05	1.50	N6	700.00	-1.05	33.00	1.00
M22	847.04	256.45	-116.82	1.50	N7	700.00	23.82	57.97	1.30
M23	1555.67	231.91	-110.00	1.50	R1	1207.0	242.23	34.00	1.80
M24	1555.67	58.33	-37.95	1.50	R2	1207.0	241.93	19.65	1.80
M25	2420.00	59.32	-34.94	1.50	R3	1207.0	580.81	132.74	3.53
M20-1	88.56	321.41	-150.00	10.00	R4	1207.0	525.73	35.00	3.53
M20-2	758.48	638.29	-150.00	10.00	R5	1207.0	885.36	153.95	6.92
M20-3	88.56	255.64	-181.87	1.50	R6	1207.0	809.00	35.00	6.92
M20-4	758.48	618.91	-163.61	1.50	R7	1207.0	1169.00	154.03	13.57
R1	1170.00	233.50	34.00	1.80	R8	1207.0	1092.51	35.00	13.57
R2	1170.00	233.30	18.41	1.80	R9	1207.0	1452.57	153.99	26.60
R3	1170.00	559.97	131.57	3.54	R10	1207.0	1376.06	35.00	26.60
R4	1170.00	507.93	35.00	3.54	R11	1207.0	1780.18	-165.66	26.60
R5	1170.00	856.41	154.54	6.96	R12	884.31	1780.18	-165.66	26.60
R6	1170.00	782.19	35.00	6.96	R13	322.69	1780.18	-165.66	26.60
R7	1170.00	1130.65	154.50	13.68	R14	322.69	1373.84	-229.76	1.80
R8	1170.00	1056.43	35.00	13.68	R15	322.69	649.07	-166.66	1.80
R9	1170.00	1361.26	140.05	25.00	R16	884.31	2151.27	-206.66	26.60
R10	1170.00	1301.31	35.00	25.00	R17	884.31	1837.02	-246.61	1.80
R11	1170.00	1665.41	-161.40	25.00	R18	884.31	1559.06	-237.66	1.80
R12	579.00	1665.41	-161.40	25.00	R19	884.31	833.42	-190.66	1.80
R13	591.00	1665.41	-161.40	25.00	R20	884.31	242.23	34.00	1.80
R14	591.00	1273.61	-227.15	1.80	R21	322.69	242.23	34.00	1.80
R15	591.00	680.09	-175.00	1.80					
R16	579.00	2140.54	-213.59	25.00					
R17	579.00	1912.79	-250.28	1.80					
R18	579.00	873.11	-198.00	1.80					
R19	579.00	233.50	34.00	1.80					
R20	591.00	233.50	34.00	1.80					
C1	430.00	-0.74	25.00	1.00					
C2	430.00	97.95	157.55	4.00					
C3	430.00	77.04	45.77	4.00					
C4	430.00	80.72	-26.00	4.00					
C5	430.00	19.27	-79.68	1.00					

Table 2 explores the optimized parameters of two cycles. However, the critical point for hydrogen occurs at approximately − 240 °C and 13 bar, where the density of liquid hydrogen is 35 kg/m3. To achieve a higher density of 76 kg/m3, especially in Case 2, higher pressures are required. The optimized operating pressures for both the hydrogen product and the hydrogen refrigerant (HF) are crucial to maintaining the desired density and optimizing the thermodynamic efficiency of the liquefaction process, ensuring efficient heat transfer and minimizing energy consumption.

### Performance analysis to base cases

A performance comparison between TCMR Case 1 and Case 2 and the base-case configurations, DCMR-OP and DCMR-H₂, is provided in Table 4. The data underscores the advantages of transitioning from a dual-cycle to a triple-cascade configuration. Under identical inlet conditions and equivalent LH₂ output, both TCMR cases achieve significant reductions in specific energy consumption (SEC) relative to the corresponding base cases.

**Table 4:** Comparison of four configurations.

Item	DCMR-OP	TCMR Case 1	DCMR-H_2_	TCMR Case 2
H_2_ feed pressure [bar]	4	4	20	20
H_2_ feed temperature [°C]	25	25	25	25
LH_2_ product pressure	21	21	19.5	47
LH_2_ temperature [°C]	-245	-245	-243	-249
LH_2_ density [kg/m^3^]	66.7	66.7	62.7	76
Cooler Outlet temperature[°C]	25	35	25	35

As indicated in Table 4, the comparison between TCMR Case 1 and DCMR-OP illustrates that both systems operate with a hydrogen feed temperature of 25 °C and a hydrogen feed pressure of 4 bar, while maintaining identical LH₂ product density, pressure, and temperature: 66.7 kg/m3, 21 bar, and − 245 °C, respectively. However, a key distinction is that the cooler outlet temperature in TCMR cases is designed to be 35 °C, representing a 28% increase over dual-cycle configurations. This increase elevates compressor power draw, although TCMR design yields a net reduction in overall power demand.

**Fig. 6:**
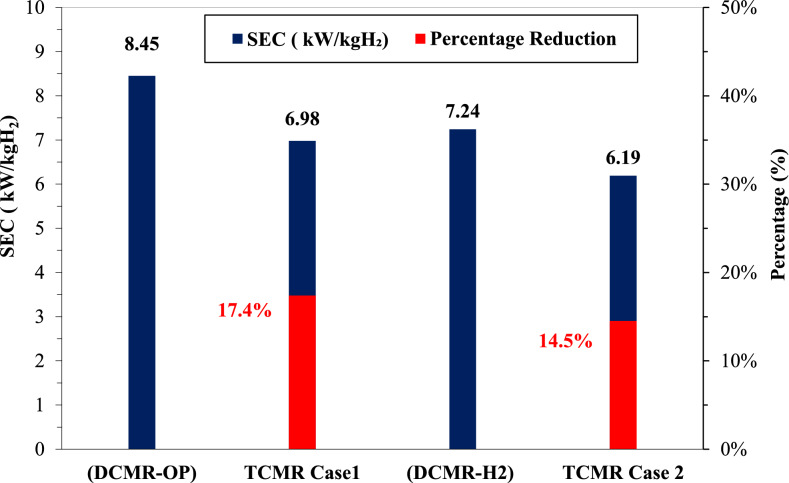
Specific energy consumption of TCMR vs. DCMR.

Similarly, comparing TCMR Case 2 with DCMR-H₂, both maintain feed conditions of 25 °C and 20 bar. However, TCMR Case 2 achieves a 17.5% increase in LH₂ density, from 62.7 to 76 kg/m3, due to its elevated product pressure (47 bar). Furthermore, the TCMR cycle attains a lower LH₂ temperature (− 249 °C vs. − 243 °C), demonstrating deeper cooling and higher density, which is critical for ultra-low-temperature hydrogen storage and transport applications. Figure 6 depicts the specific power consumption of the TCMR configurations relative to the baseline dual-cycle systems, highlighting the percentage reduction in SEC.

Figure 6 shows a remarkable reduction in specific energy consumption (SEC) of 17.4% in Case 1, achieved by utilizing the innovative TCMR cycle without ortho-para conversion reactors. Likewise, Case 2 realizes a notable 14.5% reduction in SEC while producing high-density hydrogen. These findings decisively illustrate the superior performance of the TCMR cycles compared to their dual-cycle equivalents.

### Energy analysis

**Fig. 7:**
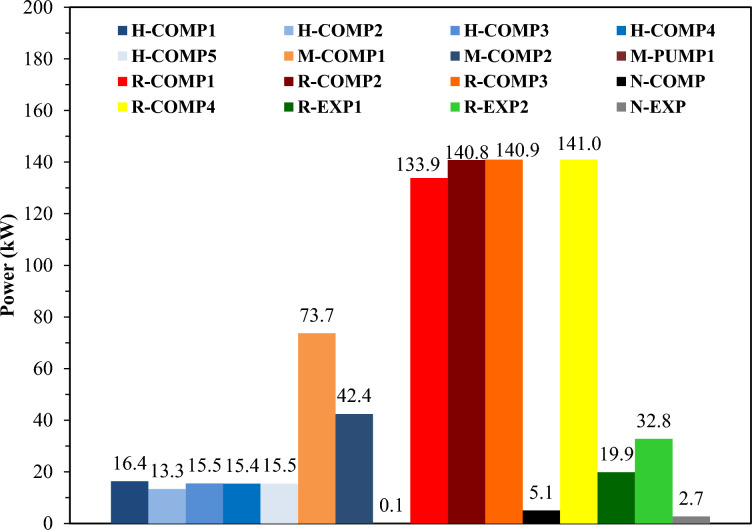
Case 1 Power consumption of each equipment and the gained power by expanders.

**Fig. 8:**
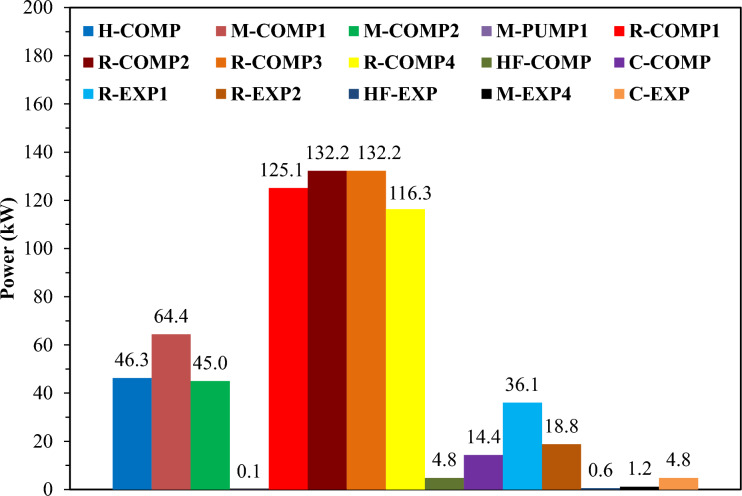
Case 2 Power consumption of equipment and the gained power by expanders.

Energy consumption is a critical indicator of system efficiency; therefore, deconstructing the energy contributions of individual equipment, including both utility machinery and energy recovery from expanders, is imperative. The notable reduction in SEC in the TCMR configurations primarily results from minimized power consumption by individual utility components. This effect is delineated in Fig. 7 (Case 1) and Fig. 8 (Case 2), which detail the power usage of individual pumps and compressors and the energy recovered by expanders.

Figure 7 demonstrates that the M-PUMP consumes the least power. The tertiary-refrigerant compressor (N-COMP) follows, drawing only 5.1 kW, a level that exerts a negligible impact on overall cycle energy demand. Conversely, compressor R-COMP-4 accounts for the highest energy consumption. The presence of the third refrigerant contributes to the reduction in R-COMP-1 power consumption. Additionally, the expander R-EXP2 recovers the greatest power, yielding 32.8 kW of energy savings.

**Fig. 9:**
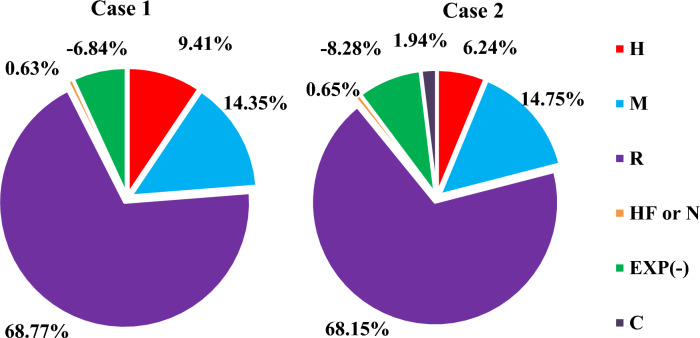
The total percentage of energy consumption cycles and saved power by expanders for TCMR Case 1 and Case 2.

Figure 8 for Case 2 also demonstrates that the M-PUMP has the lowest energy consumption, while the R-COMP-3 compressor exhibits the highest energy consumption. Additionally, the presence of a decarbonization cycle contributes to the reduction in R-COMP-1 power consumption. Furthermore, the decarbonization cycle (C) compressor operates with a moderate power consumption of 14.4 kW. Moreover, the lower power consumption of the third refrigerant in HF-COMP significantly impacts overall cycle efficiency. On the other hand, the highest power produced by an expander is from R-EXP1, at 36.1 kW. The total percentage of energy consumption and power saved by expanders for each individual cycle, including hydrogen (H), mixed refrigerant (M), mixed refrigerant (R), triple refrigerant (N) or (HF), and the percentage saved (–) for expanders is shown in Fig. 9 for Case 1 and Case 2.

Figure 9 demonstrates that the mixed-refrigerant compression loop (R) constitutes the predominant energy sink, accounting for approximately 68% of the total system consumption in both configurations. This implies that over two-thirds of the cycle’s energy demand is allocated to this loop. The mixed-refrigerant (M) compression cycle represents the second-largest consumer at 14%. Conversely, the tertiary-refrigerant circuit remains negligible, contributing just 0.6% on average. Such minimal consumption underscores the efficiency gains of introducing a third refrigerant. The hydrogen product cycle (H) accounts for 9.4% in Case 1 and 6.2% in Case 2, indicating a comparatively minor role. Expander energy recovery is moderate, with Case 2 achieving 8.2% versus 6.8% in Case 1. Finally, the Figure of Merit (FOM) improves by 4 percentage points, rising from 43 to 45% in Case 2.

### Composite curve analysis

**Fig. 10:**
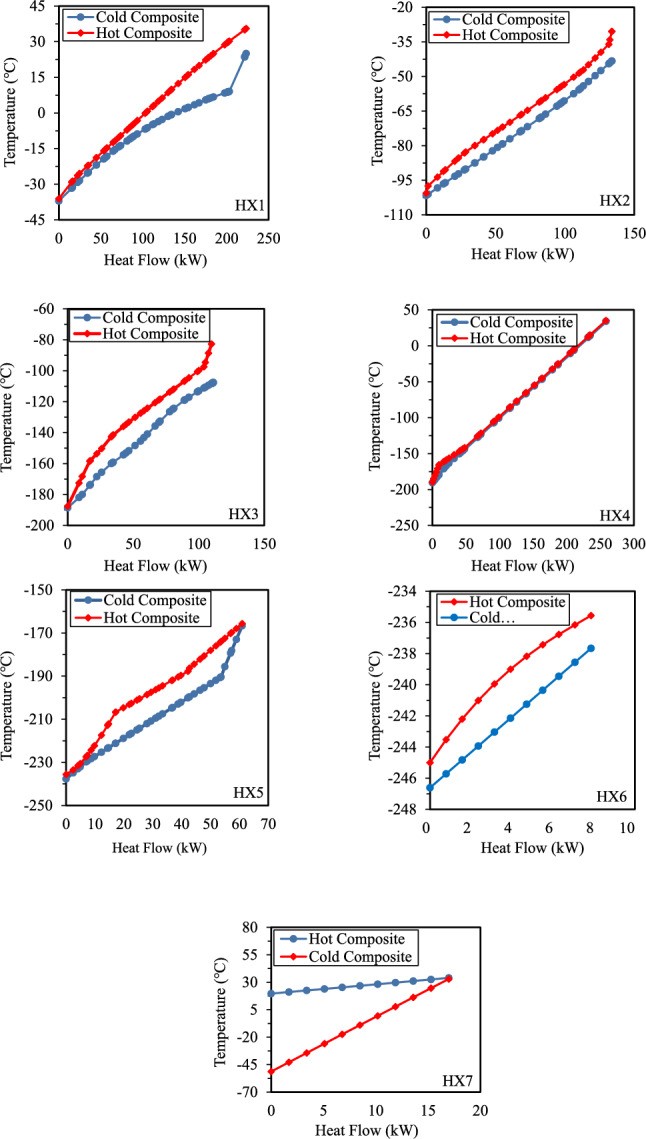
Case 1 Composite curves of HX1 to HX7.

The analysis of heat transfer in multi-stream heat exchangers is crucial for optimizing and assessing thermal performance. In TCMR Case 1, the temperatures of the cold and hot composites for each heat exchanger, as well as the heat flow for the multi-stream heat exchangers (HX1–HX7), are presented in Fig. 10.

Fig. 10 shows the temperature difference between composite curves (TDCC) for heat exchangers HX1 to HX7 in Case 1. HX1 reveals a narrow gap at lower temperatures, indicating thermodynamic feasibility, while a wider gap (0°C to 30°C) suggests variations in heat transfer effectiveness. The significant temperature drop (36.05°C) and high negative duty in pass M11–M14 confirm a strong cooling effect. HX2 and HX3 exhibit a narrow and consistent gap between the hot and cold composite curves, indicating efficient heat transfer across the exchangers. The close alignment implies minimal thermal resistance and low exergy loss, ensuring thermodynamic feasibility. Data from HX3 supports this, as pass M19–M20 shows the highest negative duty (-86.315 kW) in the hot stream, indicating substantial heat release, while M21–M22 in the cold stream presents a positive duty (111.138 kW), signifying a strong heat absorption process. The TDCC analysis of HX4 shows an exceptionally close alignment between the hot and cold composite curves, indicating efficient heat transfer. The narrow temperature gap suggests low exergy loss, making HX4 highly thermodynamically feasible. The pass data confirms strong heat exchange, particularly in R10–R11, with a high negative duty (-239.045 kW). The TDCC analysis of HX5 shows a moderate gap between the hot and cold composite curves, suggesting reasonable heat transfer efficiency but some exergy loss. The sharp slope change around mid-range heat flow indicates a phase transition. The pass data further highlights strong cooling effects, with R12–R16 experiencing a significant temperature drop (-165.66°C to -206.66°C) and a negative duty (-36.498 kW), indicating high heat rejection. Conversely, R18–R19 efficiently absorb heat (40.941 kW duty), demonstrating strong cold-side performance. Finally, the TDCC analysis of HX6 reveals a small and consistent gap between the hot and cold composite curves, indicating high heat transfer efficiency with minimal exergy loss. The curves remain closely aligned across the heat flow range. The pass data supports this, as H15–H16 (hot stream) exhibits a slight temperature drop (-235.56°C to -245.00°C) with a small duty of -7.863 kW, indicating controlled heat rejection. Meanwhile, R17–R18 (cold stream) absorbs the same amount of heat (7.863 kW duty), confirming balanced energy exchange. HX7 reveals a significant temperature gap between the hot and cold composite curves, indicating potential exergy losses. Hence, the overall TDCC analysis indicates that the choice of mixed refrigerants is reasonable and that the heat transfer performance is excellent. It should be noted that there will always be certain gaps between the composite curves of heat exchangers, which lead to entropy production. However, given the complexity of the hydrogen liquefaction process and the involvement of multiple variables, minimizing these gaps could lead to an increase in overall SEC.

**Fig. 11:**
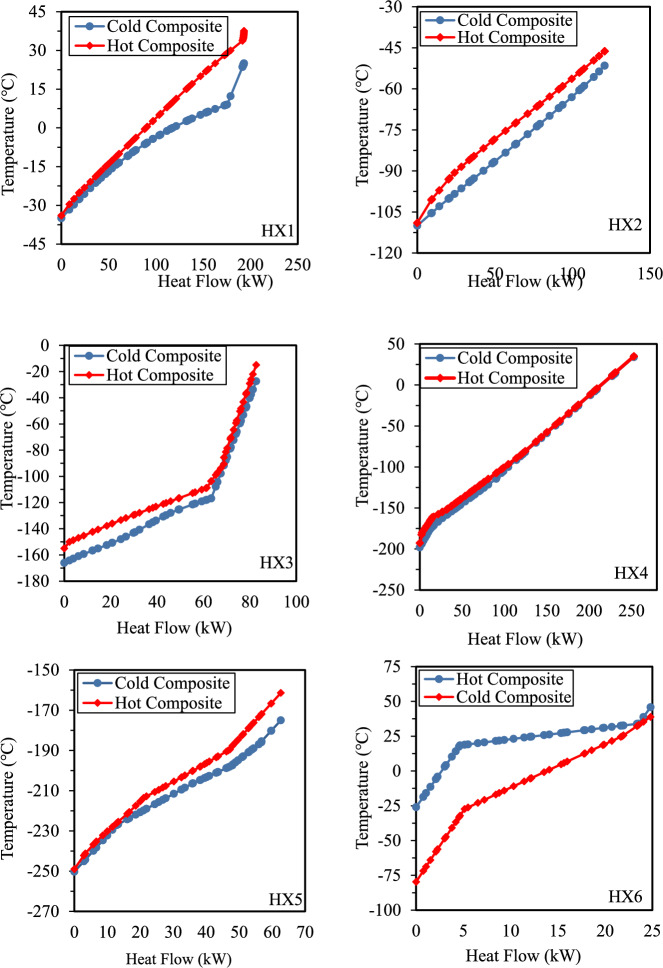
Case 2 Composite curves of HX1 to HX6.

**Fig. 12:**
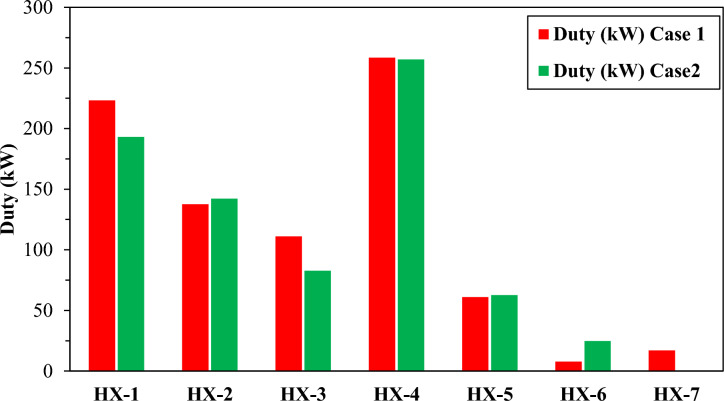
Duty for case1 and case 2.

Figure 11 shows the composite curves for heat exchangers HX1 to HX6 in Case 2, illustrating the temperature difference between composite curves (TDCC) and the energy recovery potential across the cascade stages. The TDCC analysis of HX1 shows a narrow gap at lower temperatures, indicating efficient heat transfer, while a wider gap at higher heat flows (150–200 kW) suggests a phase transition. The pass data confirms this, with M11–M14 experiencing a significant temperature drop (33.75 °C to -33.94 °C, -124.431 kW) and M25–M1 absorbing 193.19 kW, reinforcing effective energy recovery. In HX2 and HX3, the narrow, consistent gap between composite curves indicates high heat transfer efficiency. In HX3 (Case 2), the composite-curve plot displays a pronounced kink at the hand-off from Pass 2 (M19–M20, hot stream) to Pass 3 (M21–M22, cold stream). At this point, M19–M20 condenses while M21–M22 vaporizes; the opposing phase changes reverse the net heat-capacity flow and generate the curvature discontinuity. A stream-wise exergy balance (Table 5) quantifies the effect: Pass 3 creates + 78.84 kW of local exergy destruction, whereas Passes 1 and 2 remove –28.10 kW and –44.35 kW, respectively. Summed over all five passes, HX3 destroys 13.76 kW, just 0.74% of the 1.86 MW exergy supplied, giving an overall exergetic efficiency of 99.26%. HX4 and HX5 maintain efficient heat transfer, with HX4 exhibiting minimal exergy loss and strong heat rejection (-218.539 kW, R10–R11). In HX5, a moderate gap suggests reasonable efficiency, with R12–R16 experiencing a large temperature drop (-161.40 °C to -213.59 °C, -29.365 kW) and R17–R18 absorbing 28.839 kW. The TDCC analysis of HX6 reveals a larger temperature gap at lower heat flows, indicating potential exergy losses. In this heat exchanger, composite curves show distinct kinks where the net heat-capacity flow changes abruptly. This typically occurs during phase transitions, creating a critical shift in the overall enthalpy balance as Pass C3–C4 acts as the hot stream, then expands and returns to the heat exchanger in Pass C5–C1 as the cold stream. The pass data supports this, with C3–C4 showing a temperature drop (45.77 °C to -26.00 °C, -7.569 kW) and HF10–HF11 absorbing 14.324 kW, demonstrating effective heat recovery. Overall, the TDCC analysis confirms efficient heat transfer and validates the selection of mixed refrigerants. While gaps between composite curves contribute to entropy production, further reduction may increase SEC due to system complexity. Furthermore, the duty data for each multi-stream heat exchanger for Case 1 and Case 2 are shown in Fig. 12.

**Table 5:** Exergy-efficiency of multi-stream heat exchangers (Case 2).

HX	Exergy supplied (kW)	Exergetic efficiency
HX-1	1124.913	99.46%
HX-2	1354.646	99.46%
HX-3	1863.053	99.26%
HX-4	3293.417	99.54%
HX-5	3966.569	99.67%
HX-6	695.621	99.43%

Figure 12 illustrates that the heat exchanger duty varies notably between Cases 1 and 2. HX4 registers the highest duty in both scenarios, with nearly identical magnitudes. This is followed by HX1 and HX2, which display comparable duties between the two cases; however, HX1 shows a slightly lower duty in Case 2. The figure indicates that the duty in the first-stage heat exchanger, HX1 (in the precooling section), and HX4 (in the deep-cooling section), is the highest due to the temperature gradient and the phase change of the refrigerant across the heat exchangers. In addition, HX3 and HX1 in Case 2 demonstrate lower duties compared to Case 1, reflecting enhanced heat transfer in Case 2, where the temperature is lower (− 249 °C) than in Case 1 (− 245 °C).

### Exergy analysis

**Fig. 13:**
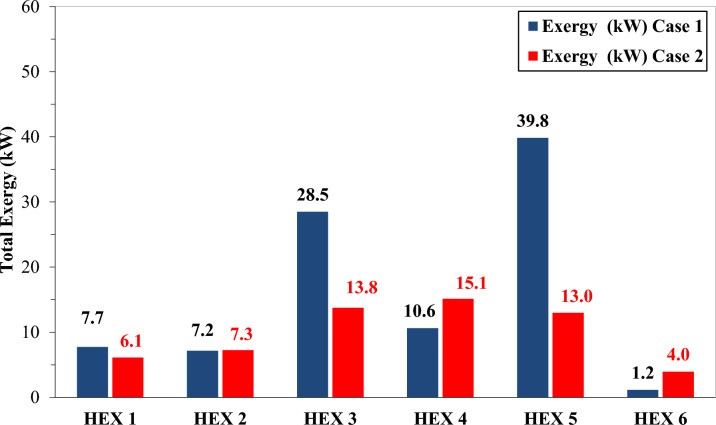
Exergy analysis of multi-stream heat exchanger Case1 and Case 2.

Exergy analysis offers critical insights into system irreversibilities, with exergy destruction serving as a primary metric for thermal inefficiency. The analysis is executed in two sequential phases. Phase one concentrates on quantifying the exergy performance of each multi-stream heat exchanger under both Case 1 and Case 2 conditions, as shown in Fig. 13.

The exergy analysis of the multi-stream heat exchangers, presented in Fig. 13, offers a quantitative comparison of exergy destruction for Case 1 and Case 2. Notably, Case 2 achieves a 51% reduction in exergy destruction in HX3 and a 67% decrease in HX5, reflecting enhanced heat recovery efficiency afforded by the optimized refrigerant flow configuration. In contrast, exergy destruction increases by 30% in HX4 and 70% in HX6, although these incremental rises remain relatively modest. Overall, Case 2 reduces total heat-exchanger exergy destruction by 37%, from 95.0 kW to 59.2 kW, underscoring its superior thermodynamic performance and lower energy input requirements compared to Case 1. Moreover, the exergy efficiency of the heat exchangers is analyzed, as presented in Table 5, particularly for Case 2. This table reports the exergy supplied and the resulting exergetic efficiency for each of the six multi-stream heat exchangers, supporting the analysis presented in Fig. 11. As shown, all exchangers demonstrate high thermodynamic efficiency, with exergetic efficiencies exceeding 99% in every case. Notably, HX3 exhibits the lowest exergetic efficiency, at 99.26%, confirming effective thermal integration despite localized irreversibility.

**Fig. 14:**
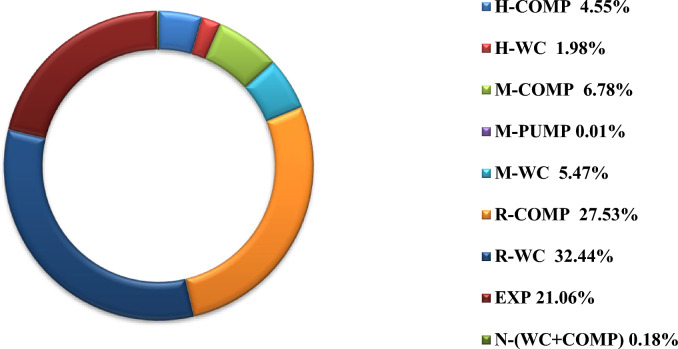
Percentage of exergy destruction for each equipment in Case1.

**Fig. 15:**
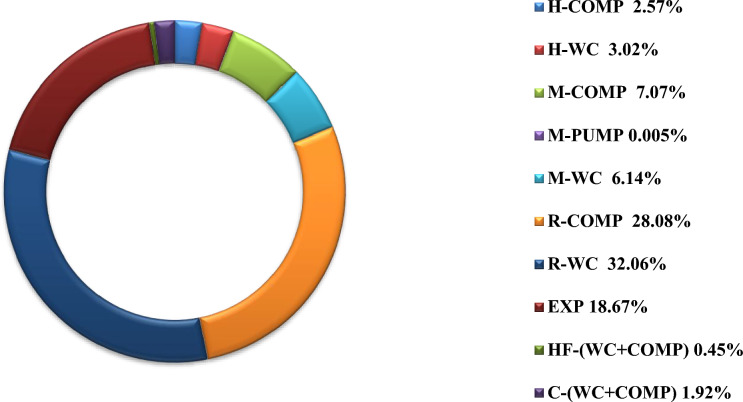
Percentage of exergy destruction for each equipment in Case 2.

In the second phase, the exergy analysis was extended to individual system equipment, including compressors, coolers, and pumps associated with each refrigerant loop and the hydrogen product cycle, as well as expanders, for both cases. Figures 14 and 15 illustrate the equipment-level percentage exergy destruction across the entire cycle.

In Fig. 14, the distribution of exergy losses across system components is evaluated. In Case 1, the largest contributors were R-WC (32.44%), R-COMP (27.53%), and EXP (21.06%), collectively accounting for over 80% of the total exergy loss.

In Fig. 15, similar trends to Case 1 are observed in Case 2. The top contributors to exergy loss are R-WC (32.06%), R-COMP (28.08%), and EXP (18.67%), collectively accounting for over 78% of the total exergy loss. These results underscore the preeminence of R-loop inefficiencies and pinpoint the expander as a key target for performance enhancement.

The cumulative exergy demand of all compressors is approximately 114.5 kW in Case 1 and 103.0 kW in Case 2. Furthermore, exergy losses associated with the tertiary refrigerant remain negligible (0.18% Case 1 and 0.45% Case 2) in both configurations. Correspondingly, the overall exergy efficiency improves from 45.2% in Case 1 to 46% in Case 2. This gain stems from decreased exergy destruction, validating the thermodynamic advantage of the hydrogen-based tertiary refrigerant integration.

### Economic analysis

**Fig. 16:**
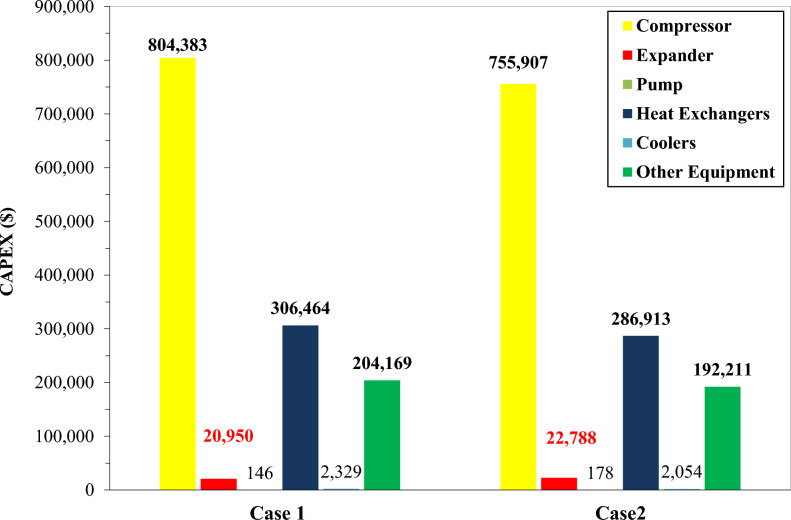
CAPEX analysis of Case1 and Case 2.

This phase of the analysis assesses the capital and operational expenditures to quantify the total cycle cost for both cases. Figure 16 depicts the CAPEX breakdown, highlighting the cost distribution across each equipment item in the proposed cycle.

**Fig. 17:**
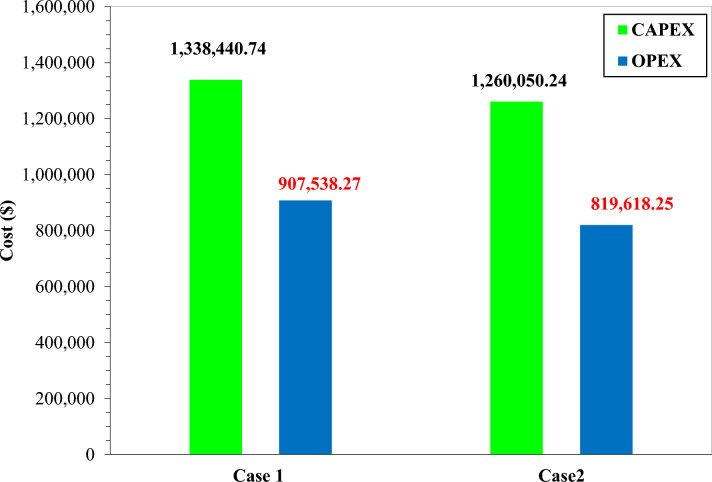
CAPEX and OPEX for both cases.

Figure 16 illustrates the CAPEX distribution for Cases 1 and 2 in the hydrogen liquefaction TCMR systems, highlighting pronounced differences in equipment capital shares. In both scenarios, compressors dominate capital expenditure at 60%, followed by heat exchangers at 23%. Auxiliary components, namely expanders, valves, pipes, pumps and others, collectively account for the remaining 17%. In Case 2, compressor costs persist as the principal cost driver at $ 755,907, representing a 6% reduction compared to Case 1. Heat exchanger CAPEX also declines by 6.38% in Case 2. However, expander costs rise from $ 20,950 in Case 1 to $ 22,788 in Case 2, an 8% increase. Figure 17 compares the aggregate CAPEX and OPEX for both configurations.

Figure 17 presents a comparative analysis of CAPEX and OPEX for Cases 1 and 2, offering critical insights into the cost framework of the hydrogen liquefaction process via TCMR systems. In Case 1, aggregate CAPEX amounts to $ 1,338,440.74, with OPEX totaling $ 907,538.27. Conversely, Case 2 achieves a CAPEX reduction to $ 1,260,050.24 and an OPEX reduction to $ 819,618.25, equivalent to 5.86% and 9.69% declines, respectively. As a result, the unit liquefaction cost in Case 2 is reduced to approximately $ 1.14/kg LH₂, compared with $ 1.26/kg LH₂ in Case 1. These outcomes underscore an enhanced cost efficiency in Case 2, driven primarily by lower operational expenditures, a factor critical to large-scale liquefaction projects.

### Environmental analysis

This section details the carbon footprint (CFP) analysis of the hydrogen liquefaction process for operational Cases 1 and 2, with emphasis on Scope 1 and Scope 2 emissions. The analysis quantifies the process’s environmental impact and assesses the effectiveness of the decarbonization strategies. Scope 1 emissions from fugitive refrigerant leaks are calculated using the mass flows and global warming potentials (GWPs) of each mixed refrigerant, as summarized in Table 6. Scope 2 emissions are derived from electricity usage and the applicable emission factor to reflect the GHG intensity of the power supply.

**Table 6:** Emissions from fugitive leakage comparison between Case1 and Case2.

Refrigerant	Component	GWP	Case 1	Case 2
			CO₂e (kg/h)	CO₂e (kg/h)
First	Methane	27	6750.8	6001.8
	Ethylene	4	1173.8	1043.6
	Ethane	2.8	679.7	604.3
	Propane	0.5	256.9	228.4
	n-Pentane	0.14	168.8	150.0
	Nitrogen	0	0	0
Second	Helium	0	0	0
	Neon	0	0	0
Third	Nitrogen/Hydrogen	0	0	0
Total CO₂e Emissions from Refrigerant (kg/h)			9,030.11	8,028.24
CO₂e Emissions from Refrigerant Leakage annually 10% (CO₂e Ton/y)			7,151.85	6,358.37
Percentage reduction in CFP in Case 2			11.1%	

Table 6 summarizes the Scope 1 carbon footprint (CFP) analysis for Cases 1 and 2, indicating an 11.1% reduction in CO₂e emissions in Case 2. Annual fugitive CO₂e emissions from refrigerant leakage decline from 7,151.85 tonnes in Case 1 to 6,358.37 tonnes in Case 2, highlighting the benefits of optimized refrigerant formulations and the integration of a CO₂-based decarbonization strategy. These results underscore the effectiveness of Case 2’s advanced design in minimizing the liquefaction process’s carbon footprint and aligning with global sustainability objectives.

Scope 2 emissions derive from purchased electricity. In Case 1, the process incurs 4,852.58 kg CO₂/yr from coal-fired generation and 2,711.25 kg CO₂/yr from natural-gas-fired generation. In Case 2, these values decrease to 2,711.25 kg CO₂/yr (coal) and 2,403.98 kg CO₂/yr (natural gas), representing an approximate 11% reduction in indirect emissions. This improvement further advances the process’s environmental performance.

A notable innovation in Case 2 is the application of captured CO₂ as a working fluid for compressor-inlet cooling, yielding two primary benefits: firstly, reduced compressor power consumption, thereby lowering CO₂ emissions by decreasing reliance on fossil-based electricity; and secondly the adoption of a natural refrigerant, enhancing system sustainability and supporting broader decarbonization initiatives.

Overall, Case 2 achieves a 22% decrease in combined Scope 1 and 2 emissions relative to Case 1 and conventional designs. This represents a significant stride towards integrating high performance with sustainable engineering practices and facilitating hydrogen’s role as a low-carbon energy carrier in accordance with SDG 13 (Climate Action). Replication of these strategies across the sector is poised to drive substantial progress in reducing GHG emissions and fulfilling global sustainability targets.

### Comparison with past research

Benchmarking the proposed TCMR cycle against contemporary literature is crucial for situating its performance within the current research, particularly concerning specific energy consumption (SEC), Figure of Merit (FOM), and LH₂ product specifications. Table 7 offers a comparative overview of the TCMR cycle and representative systems from recent publications.

**Table 7:** Comparison between TCMR and recent literature liquefaction process.

System	SEC	H_2_ Product Pressure	H_2_ Product Temperature	FOM	H_2_ Density
	kWh/kgH_2_	bar	°C	%	kg/m^3^
Large-scale hydrogen liquefaction^50^	13.58	3	-252.15	21	75.89
Closed-N₂ pre-cooling dual-path H₂ refrigeration^51^	11.4	20	-251.2	28	75
on helium reverse Brayton cycle integrating with steam methane reforming^52^	10.78	21	-252.42	42.4	76
Dual pressure Claude cycle with LN_2_ pre-cooling^53^	10.85	9.2	-252.65	36	76.48
MR pre-cooled cycle with helium expansion cycle^54^	9.7	21	-251.95	39	76.17
Large hydrogen liquefaction charge-WE-NET^55^	8.53	30	-252.75	46	76.98
Hydrogen liquefaction process with liquid system^56^	7.25	25	-253.15	53	77.88
Cyro compressed with neon process^57^	6.42	500	-213.15	34	71.59
Super critical DCMR^28^	6.87	100	-233.15	49	70.52
Super critical DPMR process with ortho-para converters^28^	7.919	100	-233.15	42.7	70.52
Conceptual design MR Brayton^58^	6.93	80	-252.95	56	79
DCMR ortho-para converters^29^	8.45	21	-245.99	21	66
DCMR with hydrogen refrigerant^30^	7.24	19.5	-243.15	42	62.72
Proposed TCMR in this paper Case 2	6.19	47	-249	45	76

Table 7 indicates that Case 2 of the proposed TCMR cycle achieves a substantial 54.4% reduction in specific energy consumption compared to conventional large-scale hydrogen liquefaction systems^53^. It also demonstrates a 26.7% reduction relative to the base-case DCMR with ortho-para converters, and a 14.5% reduction compared to the DCMR configuration employing hydrogen refrigerant.

## Conclusion

- The proposed triple-cascade mixed refrigerant (TCMR) cycles significantly reduce specific energy consumption (SEC), achieving 6.98 kWh/kgH₂ (Case 1) and 6.19 kWh/kgH₂ (Case 2), corresponding to 17.4% and 14.5% reductions compared to dual-cycle reference systems with ortho-para conversion reactors.
- Case 2 enables higher hydrogen storage density (76 kg/m3 at –249 °C) compared to Case 1 (66.7 kg/m3 at –245 °C).
- Total heat exchanger exergy destruction is reduced by 37% in Case 2, improving overall exergy efficiency from 45.2% (Case 1) to 46% (Case 2).
- Case 2 lowers both capital (5.86%) and operating expenditures (9.69%), reducing the liquefaction cost to approximately $1.14 per kg LH₂, compared to $1.26 per kg LH₂ for Case 1.
- Environmental analysis shows a 22% reduction in carbon footprint for Case 2, primarily due to CO₂ utilization for decarbonization and less power consumption.

Future research should explore renewable energy integration and compare advanced optimization methods (e.g., SQP, PSO) to further improve cycle robustness. In summary, integrating a hydrogen-based tertiary refrigerant into a triple-cascade mixed refrigerant cycle substantially improves energy efficiency, cost, and environmental performance for hydrogen liquefaction, demonstrating clear advantages over conventional designs.

## Data Availability

Data is provided within the manuscript or supplementary information files. Further simulation details and calculations are available from the corresponding author upon request.

## List of symbols

*A*: Heat transfer area, m^2^
*E*: Exergy loss, kW
*Ė*: Exergy destruction rate, kW
*e*: Mass of CO_2_ emissions, tonne
*h*: Specific enthalpy, kJ/kg
*m*: Mass flow rate, kg/h
*P*: Pressure, bar
*s*: Mass entropy, kJ/kg·K
Ṡ: Entropy generation rate, kW/K
*T*: Temperature, °C
*U*: Overall heat transfer coefficient, kW/m^2^·°C
*W*: Power, kW

## Greek symbol

*Δ*: Difference
*η*: Efficiency
*Φ*: Mass exergy or Specific exergy, kJ/kg
*ρ*: Liquid density, kg/m^3^
*τ*: Time, h
$\alpha$: CO_2_ emissions factor, gCO₂/kWh

## Subscript

act: Actual
COMP: Compressor
COOL: Cooler
EXP: Expander
ex: Exergy
feed: Raw hydrogen
gen: Generation
HX: Heat exchanger
H_2_: Hydrogen
i: Inlet
LH_2_: Liquid hydrogen
net: Net
o: Outlet
PUMP: Pump
Product: Liquid Hydrogen Product
rev: Theoretical (reversible)
ref: Reference (ambient) state
sup: Supply
Tot: Total
WC: Water cooler

## Abbreviations

CAPEX: Capital Asset Expenditure
CGH_2_: Compressed gaseous hydrogen
CFP: Carbon Footprint
C: Symbol of carbon dioxide refrigerant
COMP: Compressor
DCMR: Dual cascade mixed refrigerant cycle
EXP: Expander
FOM: Figure of merit
GHG: Greenhouse Gas
GWP: Global Warming Potential
GA: Genetic algorithm
HF: Symbol of hydrogen refrigerant
HX: Heat exchanger
He: Helium
J-B: Joule-Brayton
J-T: Joule-Thomson
LMTD: Logarithmic mean temperature difference
LNG: Liquefied natural gas
MIX: Mixer
MR: Mixed refrigerant for base case
M: Symbol of Mixed refrigerant 1
R: Symbol of Mixed refrigerant 2
N: Symbol of Nitrogen refrigerant
OPEX: Operational expenditure
OP: Ortho-parahydrogen conversion
PC: Purchased cost
PR: Peng-Robinson
PSO: Particle Swarm Optimization
SEC: Specific energy consumption
SDG: Sustainable Development Goal
SQP: Sequential quadratic programming
TDCC: Temperature difference between composite curves
TCMR: Triple Cascade Mixed Refrigerant
