# Correction: Comparative study of advanced hydrogen liquefaction using triple cascade mixed refrigerant cycles with integrated energy exergy economic and environmental analysis

**DOI:** 10.1038/s41598-026-36689-7

**Published:** 2026-01-21

**Authors:** M. Shawky Ismail, M. Abd ElSalam ElSeuofy, Abd ElHamid Attia, Wael M. El-Maghlany, Mohamed ElHelw

**Affiliations:** Mechanical Engineering Department, Faculty of Engineering, Alexandria University, Alexandria, 21544 Egypt

Correction to: *Scientific Reports* 10.1038/s41598-025-14258-8, published online 01 September 2025

The original version of this Article contained an error in the spelling of the author Wael M. El-Maghlany, which was incorrectly given as Wael ElMaghlany.

The original Article has been corrected.

